# Factores asociados con la presencia de endoparásitos y ectoparásitos en perros domiciliados de la zona metropolitana de Toluca, México

**DOI:** 10.7705/biomedica.6013

**Published:** 2021-12-15

**Authors:** Elizabeth Lara-Reyes, Israel A. Quijano-Hernández, Roger I. Rodríguez-Vivas, Javier Del Ángel-Caraza, José Simón Martínez-Castañeda

**Affiliations:** 1 Hospital Veterinario para Pequeñas Especies, Facultad de Medicina Veterinaria y Zootecnia, Universidad Autónoma del Estado de México, Toluca, México Universidad Autónoma del Estado de México Universidad Autónoma del Estado de México Toluca Mexico; 2 Laboratorio de Parasitología, Campus de Ciencias Biológicas y Agropecuarias, Facultad de Medicina Veterinaria y Zootecnia, Universidad Autónoma del Estado de México, Yucatán, México Universidad Autónoma del Estado de México Universidad Autónoma del Estado de México Yucatán Mexico; 3 Centro de Investigación y Estudios Avanzados en Salud Animal, Facultad de Medicina Veterinaria y Zootecnia, Universidad Autónoma del Estado de México, Toluca, México Universidad Autónoma del Estado de México Universidad Autónoma del Estado de México Toluca Mexico

**Keywords:** zoonosis/epidemiología, Giardia, Ancylostoma, Toxocara canis, Ctenocephalides, México, Zoonoses/epidemiology, Giardia, Ancylostoma, Toxocara canis, Dipylidium caninum, Ctenocephalides, México

## Abstract

**Introducción.:**

Los endoparásitos y ectoparásitos en perros son de distribución mundial. La estrecha relación entre los perros y el hombre implica un riesgo de transmisión de parasitosis zoonóticas, por lo cual es necesario conocer las especies que parasitan a los perros de esta zona y determinar los factores asociados.

**Objetivos.:**

Estimar la prevalencia de endoparásitos y ectoparásitos, identificarlos en perros domiciliados de la zona metropolitana de Toluca, México, y determinar la prevalencia de *Dipyilidium caninum* en pulgas del género *Ctenocephalides* spp.

**Materiales y métodos.:**

Se recolectaron muestras de 402 perros que fueron llevados a consulta en cuatro hospitales de referencia de Toluca. En el diagnóstico de endoparásitos, se utilizaron las técnicas coproparasitoscópicas de frotis directo, flotación y sedimentación; además, se recolectaron ectoparásitos para su identificación taxonómica. Por último, la detección de *D. caninum* en pulgas se hizo mediante la reacción en cadena de la polimerasa (PCR).

**Resultados.:**

El 37,2 % de los perros resultó positivo para endoparásitos. Los géneros o especies identificados fueron *Toxocara* spp., *Giardia* spp., *Ancylostoma* spp., *Cystoisospora* spp., *D. caninum*, *Taenia* spp. y *Trichuris vulpis*. Se determinó una prevalencia de ectoparásitos de 13,13 %. Se identificaron pulgas de las especies *Ctenocephalides felis* y *C. canis*, en tanto que solo un animal presentó parasitosis por *Rhipicephalus sanguineus* y otro por *Trichodectes canis*. La prevalencia de *D. caninum* en pulgas fue del 9,5 %.

**Conclusión.:**

La prevalencia de endoparásitos fue de 37,2 % y, la de ectoparásitos, de 13,1 %. Por primera vez en México se hizo un análisis de endoparásitos y ectoparásitos en una misma población de perros, así como el diagnóstico molecular de *D. caninum*.

## Abstract (en)

**Introduction:**

Endoparasites and ectoparasites in dogs are of global distribution. The close relationship between dogs and man implies a risk for the transmission of zoonotic parasites. Therefore, it is necessary to determine the parasites hosted by dogs in specific areas and the factors associated with their presence.

**Objectives:**

To identify and to estimate the prevalence of endoparasites and ectoparasites in domiciled dogs in the Metropolitan area of Toluca, México, and the prevalence of *D. caninum* in fleas of the genus *Ctenocephalides* spp.

**Materials and methods:**

We collected samples from 402 domiciled dogs in four reference hospitals in the area in Toluca. We diagnosed endoparasites using direct smear, flotation, and sedimentation techniques and we performed the taxonomic identification of ectoparasites. Finally, the molecular diagnosis of *D. caninum* in fleas was made using the polymerase chain reaction technique (PCR).

**Results:**

A total of 37.2% of dogs were positive for endoparasites; the genera or species identified were *Toxocara* spp., *Giardia* spp., *Ancylostoma* spp., *Cystoisospora* spp., *D. caninum*, *Taenia* spp., and *Trichuris vulpis*; the prevalence of ectoparasites was 13.13%. We identified fleas of the species *Ctenocephalides felis*, *Ctenocephalides canis*; only one animal was parasitized with Rhipicephalus sanguineus and another one with *Trichodectes canis*; the prevalence of *D. caninum* in fleas was 9.5%.

**Conclusion:**

The prevalence of endoparasites was 37.2% while that of ectoparasites was 13.1%; this is the first analysis of endoparasites and ectoparasites conducted in the same population of dogs in México together with the molecular diagnosis of *D. caninum* in fleas.

En la actualidad, los perros tienen un papel importante en la sociedad como animales de compañía, para guardia, protección y rescate, así como de apoyo en terapia ocupacional ^1^; sin embargo, pueden ser huéspedes definitivos de parásitos zoonóticos como *Giardia* spp., *Ancylostoma* spp., *Toxocara canis*, *Echinococcus* spp., *Dipylidium caninum*, *Strongyloides* spp., entre otros ^2^^,^^3^. Además, se consideran esenciales en la epidemiología de las enfermedades transmitidas por vectores, como mosquitos, garrapatas, pulgas y flebótomos, entre las cuales cabe mencionar babesiosis, anaplasmosis, leishmaniasis, borreliosis, ricketsiosis y dipilidiasis ^4^.

En diversos estudios se han reportado prevalencias variables de endoparásitos en perros domiciliados: en Estados Unidos, se encontró una prevalencia nacional del 12,5 % ^5^, en Austria, se reporta un 6 % ^6^, en tanto que en Brasil, una mayor, de 43 % ^7^. En México, las prevalencias son variables. En Villahermosa, Tabasco, se ha registrado una prevalencia de 26,50 % ^8^, en Ciudad de México, de 21,3 % y, en Mérida, de 46,2 % ^9^^,^^10^. Esta variación se debe a las características climáticas y las condiciones socioeconómicas de cada región ^11^. A pesar de esta gran diversidad en las prevalencias reportadas, *Toxocara* spp., *Giardia* spp. y *Ancylostoma* spp. tienen la mayor distribución y afectan a perros de distintos países ^11^.

Entre los ectoparásitos que afectan a los perros, las garrapatas y las pulgas ocasionan los principales daños y pueden transmitirles agentes patógenos ^12^^-^^14^. En México, las pulgas *Ctenocephalides felis* y *Ct. canis*^15^ y la garrapata *Rhipicephalus sanguineus* son los ectoparásitos que más frecuentemente afectan a perros de áreas urbanas y rurales ^16^^,^^17^.

En un estudio reciente en el sureste de México, se reportó que los perros pueden estar parasitados con nueve diferentes especies de garrapatas, entre ellas, *Ixodes near affinis*, *Amblyomma mixtum*, *A. sabanerae*, *A. parvum*, *A. ovale*, *A. auricularium*, *A. maculatum*, *Dermacentor nitens* y *R. sanguineus*^18^. A pesar de estos registros, en el centro del país (Estado de México) hay pocos estudios sobre los endoparásitos y ectoparásitos que afectan a los perros y el riesgo de transmisión a la población humana.

Dados los serios problemas de salud que los endoparásitos y ectoparásitos ocasionan a los perros, y que algunos de ellos pueden ser zoonóticos, es imperante conocer su diversidad y abundancia en áreas geográficas específicas, para establecer medidas tendientes a reducir su impacto negativo en los animales y el riesgo de transmisión a la población humana.

Por estas razones, el objetivo del presente estudio fue estimar la prevalencia de endoparásitos y ectoparásitos, identificarlos en perros domiciliados de la zona metropolitana de Toluca, México, y determinar los factores asociados con la presencia de cada especie parasitaria.

## Materiales y métodos

### Lugar de estudio

El estudio se hizo en cuatro hospitales veterinarios de municipios de la zona metropolitana de Toluca, México, (Metepec, San Mateo Atenco, Toluca y Zinacantepec). La población humana estimada en esta zona es de 1,85 millones de habitantes, su superficie es de 1.991 km^2^ y la densidad urbana de 67,1 habitantes/ha. Su altitud promedio es de 2.660 msnm, tiene un clima templado (promedio anual de 15 °C) y una humedad relativa promedio de 70 % ^19^.

### Tamaño de muestra y población de estudio

Mediante la fórmula de Cochran ^20^, se calculó el tamaño de la muestra considerando una precisión de 0,05, con un nivel de confianza del 95 %, una prevalencia esperada del 20 % ^9^ y una población considerada de aproximadamente 308.333 perros, según lo sugerido por la WHO-WSPA de un perro por cada seis habitantes ^21^. El número de animales por muestrear fue de 198 perros domiciliados, cantidad que se duplicó para el estudio considerando dos épocas del año: de lluvias (junio a octubre de 2016) y seca (noviembre de 2016 a mayo de 2017). Se hizo un muestreo estratificado de perros según la densidad poblacional por municipio. Ninguno de los perros estudiados recibió desparasitación interna o externa en los 60 días previos al muestreo.

### Toma de muestras

En la consulta se les informó a los propietarios sobre los objetivos del estudio y se solicitó su consentimiento informado para la toma de muestras a sus mascotas. Las personas proporcionaron la siguiente información de los perros: edad, sexo, raza, si tenían acceso a la calle y si permanecían dentro o fuera de la casa. En la inspección general de los animales, se obtuvieron los siguientes datos: condición corporal determinada mediante la escala de Laflamme (se catalogaron como deficientes los grados 1 y 2, y como no deficientes, los grados 3, 4 y 5) ^22^, consistencia de las heces (firme, diarrea), y otras manifestaciones de enfermedad intestinal (dolor abdominal, borborigmo o líquido en asas intestinales).

A cada perro se le tomó un hisopado rectal para el análisis con la técnica directa en fresco y una impresión de la zona perianal con la ayuda de una cinta adhesiva transparente ^23^, Además, se solicitó a los propietarios que recolectaran muestras de heces en tres días consecutivos para el análisis coproparasitoscópico.

Se recolectaron las garrapatas y los piojos mediante desprendimiento manual con ayuda de pinzas y un guante de inspección, y las pulgas, con un peine de cepillado y obtención de especímenes ^24^. Los ectoparásitos se colocaron en etanol al 70 % y se mantuvieron en refrigeración a 4 °C hasta su identificación taxonómica.

### Pruebas de laboratorio

Las heces de los perros se procesaron en el laboratorio clínico del Hospital Veterinario para Pequeñas Especies de la Facultad de Medicina Veterinaria y Zootecnia de la Universidad Autónoma del Estado de México, usando las siguientes técnicas de diagnóstico de endoparásitos: estudio coproparasitoscópico directo, técnica de flotación en solución de sulfato de cinc y Sheather, técnica de sedimentación y test de Graham ^23^. Las pulgas, garrapatas y piojos se identificaron de acuerdo con su morfología y siguiendo las llaves taxonómicas correspondientes ^4^^,^^25^.

Se consideraron como positivas para endoparásitos aquellas muestras con huevos, ooquistes, larvas, adultos o proglótidos detectados mediante alguna de las técnicas coproparasitoscópicas mencionadas. De los animales con ectoparásitos, se recolectaron e identificaron especímenes de pulgas, garrapatas o piojos.

Además, en la unidad de diagnóstico molecular de la Facultad de Medicina Veterinaria y Zootecnia de la Universidad Autónoma de Yucatán, se hizo el diagnóstico molecular de *D. caninum* en las pulgas obtenidas en los muestreos, siguiendo la siguiente metodología.

*Extracción de ADN*. Las pulgas obtenidas de cada paciente fueron analizadas de manera individual para identificar su especie; si un perro presentaba más de dos pulgas, se hacía una mezcla de dos a tres de ellas, de tal manera que se obtuvieran muestras de pulgas individuales y muestras de mezclas de la misma especie. Las pulgas se retiraron del alcohol y se lavaron con agua destilada para luego almacenarlas a -70 °C durante 12 horas. Posteriormente, con la ayuda de un pistilo de plástico estéril, se maceraron y se extrajo el ADN con el estuche Quiagen DNAeasy Blood&Tissue® siguiendo las instrucciones del fabricante. Las muestras se conservaron a -20 °C hasta el momento en que se hizo la PCR.

*Amplificación de ADN y electroforesis*. Para el diagnóstico de *D. caninum* en las pulgas, se utilizó la región *28S rDNA* del genoma del parásito, cuyo tamaño esperado era de 653 pb. Se utilizaron los siguientes iniciadores para el diagnóstico: C28S-1R:5-CACATTCAACGCCCGACTCCTGTAG-3 y DC28S- 1F:5-GCATGCAATCAAAGGGTCCTACG-3 ^26^. El volumen final de la mezcla para la PCR fue de 25 μl, que contenía 12,5 μl de Gotaq Green Master Mix®, 2,5 μl de cada iniciador, 5 μl de H_2_O y 5 μl de cada muestra de ADN.

Las condiciones del ciclo fueron: 95 °C por 15 minutos, 40 ciclos a 94 °C por 30 segundos, 56 °C por 30 segundos, 72 °C po 30 segundos y 72 °C por 10 minutos ^27^. La electroforesis de tos productos de Ια PCR se llevó a cabo en gel de agarosa al 1,5 % teñido con bromuro de etidio. Se utilizaron 7 μl del marcador de peso molecular Thermo Scientific® para 1.000 pb y, para el control positivo y las muestras, se utilizó un volumen de 10 μl a un voltaje de 104 v y 110 mA durante 45 minutos.

### Análisis estadístico

Se determinó la prevalencia general y por especie de parásito. Inicialmente, se realizó la prueba de ji al cuadrado con el programa Prisma Graphpad®, considerando como variables dependientes a los endoparásitos con mayor prevalencia: *Toxocara* spp., *Giardia* spp., *Ancylostoma* spp., *D. caninum* y *Cystoisospora* spp., y como variables independientes de cada parásito, al municipio (Metepec, San Mateo Atenco, Toluca o Zinacantepec), la estacionalidad (lluvias, sequías), la edad (un año o meses y más de un año), el sexo (machos, hembras), la raza (mestizos, raza pura), el salir a la calle (sí, no), la convivencia con otros animales (sí, no), la estancia en exteriores o interiores, la condición corporal (deficiente o no deficiente), las heces (firme, diarrea), y otros signos clínicos asociados con enfermedad intestinal (sí, no) o presencia de pulgas (sí, no).

Εl análisis de ji al cuadrado en ectoparásitos se hizo unicamente en pulgas porque solo se encontró un animal positivo para garrapatas y otro para piojos. Además de las variables independientes consideradas para los endoparásitos, se consideraron también el prurito (positivos, negativos) y las lesiones en piel (positivos, negativos).

El análisis de regresión logística se hizo con el programa Sigma Plot.11®. Para cada especie o género de parásito, se consideraron aquellas variables independientes con una p menor de 0,2 en la prueba de ji al cuadrado. Se calcularon la razón de momios (*Odds Ratio*, OR) e intérvalos de confianza de 95 %, y se determinaron los factores asociados con cada especie o género de endoparásitos y ectoparásitos (factores que obtuvieron un valor de p<0,05) ^2^^,^^28^.

Todos los procedimientos descritos fueron aprobados por el Comité Interno para el Cuidado de Ios Animales de Laboratorio - Docencia, Investigación, Servicio y Producción de la FMVZ-UAEM (CICUAL-DISP 17ABRIL12:30)

## Resultados

Durante los meses de junio de 2016 a mayo de 2017, se evaluaron 403 perros, de los cuales el 37,2 % (IC_95%_ 32,6-42,0) (150/403) resultó positivo, por lo menos, para un género o especie de parásito gastrointestinal. En el municipio de Metepec, se observó una prevalencia de 32,2 % (IC_95%_ 22,0-44,6) (20/62), en San Mateo Atenco, de 41,4 % (IC_95%_ 27,8-56,6) (17/41), en Toluca, de 37,6 % (IC_95%_ 31,8-43,7) (94/250) y, en Zinacantepec, de 38 % (IC_95%_ 25,951,8) (19/50), No se observó diferencia estadística entre los municipios.

Se identificaron siete géneros o especies de parásitos gastrointestinales. *Toxocara* spp. Presentó la prevalencia más alta, con 16,6 % (67/403), seguido por *Giardia* spp., con 13,4 % (54/403), *Ancylostoma* spp., con 9,2 % (37/403), *D. caninum*, con 4,7 % (19/403), *Cystoisospora* spp., con 4,7 % (19/403), *Taenia* spp., con 0,7 % (3/403), y *T. vulpis*, con 0,2 % (1/403). Se detectó multiparasitosis en 10,1 % (41/403) de los perros, de los cuales 7,9 % (32/403) presentó dos parásitos y, 2,2 % (9/403), tres parásitos. La asociación más frecuente fue entre un protozoario y un nematodo (cuadro 1).

*Toxocara* spp., *Giardia* spp., *Ancylostoma* spp., *D. caninum*, *Taenia* spp. y *T. vulpis*, presentaban potencial zoonótico. Todos los perros con multiparasitosis presentaban, por lo menos, una especie zoonótica.

Las variables con un valor de p<0,2 en la prueba de ji al cuadrado incluidas en el análisis de regresión logística, se presentan por género o especie, así: *Toxocara* spp.(cuadro 2), *Giardia* spp. (cuadro 3), *Ancylostoma* spp. (cuadro 4), *D. caninum* (cuadro 5) y *Cystoisospora* spp. (cuadro 6). El municipio, la estacionalidad, la convivencia con otros animales y el salir a la calle, no fueron estadísticamente significativos para ninguno de estos parásitos.

Los animales de un año o menores tuvieron mayor riesgo de infección con *Toxocara* spp. (cuadro 2). Aquellos con una edad de un año o menos, con estancia en exteriores, con diarrea o con otros signos clínicos asociados con enfermedad intestinal, tuvieron mayor riesgo de infección con *Ancylostoma* spp. (cuadro 3). Los perros con diarrea, con signos clínicos asociados con enfermedad intestinal y presencia de pulgas, tuvieron un mayor riesgo de infección por *D. caninum* (cuadro 4). Los perros con diarrea tuvieron mayor riesgo de infección por *Giardia* spp. (cuadro 5) y aquellos con diarrea y signos clínicos asociados con enfermedad intestinal tuvieron mayor probabilidad de infección por *Cystoisospora* spp. (cuadro 6).

De los 403 perros estudiados, el 13,15 % (IC_95%_ 10,2 %-16,8 %), (53/403) fueron positivos a ectoparásitos. En el municipio de Metepec se observó una prevalencia de 11,2 % (IC_95%_ 5,6 %-21,5 %) (7/62), en San Mateo Atenco, de 17,1 % (IC_95%_ 8,5 %-31,3 %) (7/41), en Toluca, de 12 % (IC_95%_ 8,5%-16,6%) (30/250) y, en Zinacantepec, de 18 % (IC_95%_ 9,8 %-30,8 %) (9/50). No se observaron diferencias estadísticamente significativas (p>0,05) entre los municipios.

**Cuadro 1:** Asociaciones parasitarias y frecuencia de multiparasitosis

Parasitosis doble	Frecuencia
Nematodo más protozoo	
*Ancylostoma* spp. más *Cystoisospora* spp.	6,2 % (2/32)
*Ancylostoma* spp. más *Giardia* spp.	12,5 % (4/32)
*Toxocara* spp más *Cystoisospora* spp.	12,5 % (4/32)
*Toxocara* spp. más *Giardia* spp.	18,7 % (6/32)
Nematodo más cestodo	
*Ancylostoma* spp. más *D. caninum*	6,2 % (2/32)
*Ancylostoma* spp. más *Taenia* spp.	3,1 % (1/32)
*Toxocara* spp. más *D. caninum*	6,2 % (2/32)
Nematodo más nematodo	
*Ancylostoma* spp. más *Toxocara* spp.	21,9 % (7/32)
*Toxocara* spp. más *T. vulpis*	3,1 % (1/32)
Cestodo más protozoo	
*D. caninum* más *Giardia* spp.	9,4 % (3/32)
Total	7,9 % (32/403)
Parasitosis triple		
Nematodo más protozoos	
*Ancylostoma* spp. más *Cystoisospora* spp. más *Toxocara* spp.	11.1 % (1/9)
*Ancylostoma* spp. más *Giardia* spp. más *Toxocara* spp.	22.2 % (2/9)
Nematodo más cestodo más protozoo	
*Ancylostoma* spp. más *D. caninum* más *Giardia* spp.	22.2 % (2/9)
*Toxocara* spp. más *D. caninum* más *Giardia* spp.	11.1 % (1/9)
Nematodo más cestodos	
*Ancylostoma* spp. más *D. caninum* más *Toxocara* spp.	22.2 % (2/9)
Nematodo más protozoos	
*Toxocara* spp. más *Cystoisospora* spp. más *Giardia* spp.	11.1 % (1/9)
Total	2.2 % (9/4O3)

**Cuadro 2:** Prevalencia y resultados de regresión logística (OR, IC95%, p) de *Toxocara* spp. para conocer sus factores asociados

Variables	Total	Positivos	Prevalencia	OR	IC_95%_	p
Edad						
Adulto	219	7	3,2	1		
<1 año	184	60	32,6	19,71	4,51-86,03	<0,001*
Raza						
Raza pura	309	44	14,2	1		
Mestizos	94	23	24,5	1,02	0,41-2,55	0,956
Sale a la calle						
No	219	37	16,9	1		
Sí	184	30	16,3	0,99	0,42-2,30	0,988
Condición corporal						
No deficiente	299	39	13,0	1		
Deficiente	104	28	26,9	1,09	0,37-3,19	0,874
Heces						
Firme	250	23	9,2	1		
Diarrea	153	44	28,7	0,58	0,23-1,43	0,237
Cuadro clínico asociado						
con enfermedad intestinal						
Sí	272	26	9,5	1		
No	131	41	31,3	0,31	0,08-1,13	0,078

* Estadísticamente significativo

**Cuadro 3:** Prevalencia y resultados de regresión logística (OR, IC_95%_, p) de *Giardia* spp. para conocer sus factores asociados

Variables	Total	Positivos	Prevalencia	OR	IC_95%_	p
Edad						
Adulto	219	24	10,9	1		
<1 año	184	30	16,3	1,41	0,76-2,64	0,272
Sale a la calle						
No	219	31	14,1	1		
Sí	184	23	12,5	0,85	0,46-1,59	0,626
Estancia						
Interiores	210	23	10,9	1		
Exteriores	193	31	16,1	1,21	0,64-2,26	0,544
Heces						
Firme	250	23	9.,2	1		
Diarrea	153	31	20,2	3,10	1,63-589	<0,001*

* Estadísticamente significativo

**Cuadro 4:** Prevalencia y resultados de regresión logística (OR, IC95%, P) de *Ancylostoma* spp. para conocer sus factores asociados

Variables	Total	Positivos	Prevalencia	OR	IC_95%_	p
Edad						
Adulto	219	14	6,4	1		
<1 año	184	23	12,5	2,04	1,10-3,77	0,023*
Sexo						
Hembras	182	12	6,6	1		
Machos	221	25	19,1	1,13	0,61-2,10	0,682
Raza						
Raza pura	309	19	6,1	1		
Mestizos	94	18	19,1	0,63	0,30-1,33	0,232
Sale a la calle						
No	219	9	4,1	1		
Sí	184	28	16,0	1,34	0,72-2,48	0,345
Estancia						
Interiores	210	12	5,7	1		
Exteriores	193	25	14,9	2,00	1,O8-3,73	0,027*
Condición corporal						
No deficiente	299	17	5,7	1		
Deficiente	104	20	19,2	0,99	0,47-2,08	0,996
Heces						
Firme	250	11	4,4	1		
Diarrea	153	26	17,0	1,89	1,00-3,57	0,048*
Cuadro clínico asociado con						
enfermedad intestinal						
Sí	272	13	4,8	1		
No	131	24	18,3	6,39 3,76-12,80 <0,001*		

* Estadísticamente significativo

**Cuadro 5:** Prevalencia y resultados de regresión logística (OR, IC_95%_, P) de *Dipylidium caninum* para conocer sus factores asociados

Variables	Total	Positivos	Prevalencia	OR	IC_95%_	p
Raza						
Raza pura	309	12	3,9	1		
Mestizos	94	7	7,4	1,09	0,47-2,52	0,841
Estancia						
Interiores	210	5	2,4	1		
Exteriores	193	14	7,2	1,74	0,81-3,74	0,155
Condición corporal						
No deficiente	299	11	3,6	1		
Deficiente	104	8	7,7	0,82	0,33-2,02	0,676
Heces						
Firme	250	6	2,4	1		
Diarrea	153	13	8,5	2,57	1,17-5,61	0,018*
Cuadro clínico asociado						
con enfermedad intestinal						
Sí	272	6	2,2	1		
No	131	13	9,9	4,80 2,25-10,24		<0,001*
Pulgas						
Sí	351	9	2,5	1		
No	52	10	19,2	3.32	1,53-7,20	0,002*

* Estadísticamente significativo

**Cuadro 6:** Prevalencia y resultados de regresión logística (OR, IC*95%*, p) de *Cystoisospora* spp. para conocer sus factores asociados

Variables	Total	Positivos	Prevalencia	OR	IC_95%_	p
Edad						
Adulto	219	5	2,3	1		
<1 año	184	14	7,6	1,60	0,03-0,10	0,,102
Raza						
Raza pura	309	17	5,5	1		
Mestizos	94	2	2,1	1,35	0,91-2,84	0,347
Heces						
Firme	250	4	1,6	1		
Diarrea	153	15	9,8	2,27	0,71-2,56	0,005*
Cuadro clínico asociado						
con enfermedad intestinal						
Sí	272	5	1,8	1		
No	131	14	10,7	4,98	1,28-4,01	<0,001*

* Estadísticamente significativo

La prevalencia general de perros con pulgas fue de 12,9 % (52/403) y se recolectaron entre 1 y 8 pulgas por animal. En estos 52 perros, se recolectaron 145 pulgas, de las cuales el 56,6 % (82/145) era *Ct. felis* y el 43,3 % (63/145), *Ct. canis*.

Solamente un perro resultó positivo para garrapatas (0,24 %; 1/403), con seis especímenes de *R. sanguineus*, y un perro (0,24 %; 1/403) fue positivo para *T. canis*; se recolectaron tres especímenes de este piojo.

**Figura 1:**
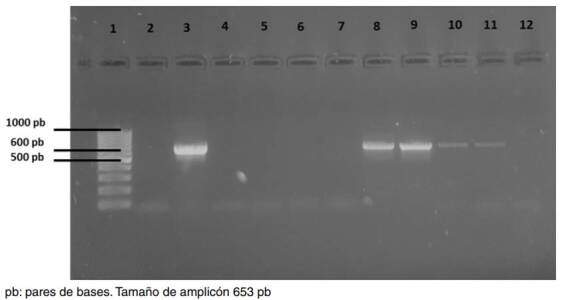
Gel de agarosa al 1,5 % teñido con bromuro de etidio para identificar la presencia de *Dipyilidium caninum* en pulgas de perros domiciliados de la zona metropolitana de Toluca, México. Carril 1: peso molecular, carril 2: control negativo, carril 3: control positivo, carriles 4 a 7: muestras negativas, carriles 8, 9 y 11: muestras positivos de *Ct. felis*, y carril 10: muestra positiva de *Ct. canis*.

Las variables con significación estadística (p<0,05) para pulicosis en el modelo de regresión logística, fueron la condición corporal, el prurito y las lesiones en piel (cuadro 7). Se extrajo ADN de 52 muestras de pulgas, de las cuales 33 correspondieron a la especie *Ct. felis* y 19 a *Ct. canis*. En el cuadro 7 se especifica la cantidad de muestras obtenidas de pulgas individuales o en conjuntos por especie. El 9,6 % (5/52) de las muestras resultaron positivas para *D. caninum* en la PCR (figura 1). *Ct. felis* fue la especie de mayor prevalencia, con 7,7 % (4/52), en tanto que *Ct. canis* tuvo una prevalencia de 1,9 % (1/52) (cuadro 8).

**Cuadro 7:** Prevalencia y resultados de regresión logística (OR, IC_95%_, P) de pulgas para conocer factores asociados

Variables	Total	Positivos	Prevalencia	OR	IC_95%_	p
Edad						
Adulto	219	27	12,3	1		
<1 año	184	46	25,0	1,39	0,87-2,22	0,161
Sexo						
Hembras	309	35	11,3	1		
Machos	94	18	19,1	1,12	0.54-2.30	0,756
Raza						
Raza pura	184	18	9,8	1		
Mestizos	219	35	15,9	1,65	0,84-3,26	0,144
Sale a la calle						
No	168	17	10,1	1		
Sí	235	36	15,3	1,55	0,81-2,97	0,181
Estancia						
Interiores	210	20	9,5	1		
Exteriores	193	33	17,1	1,50	0,76-2,94	0,233
Condición corporal						
No deficiente	294	27	9,2	1		
Deficiente	104	26	25,0	2,43	1,21-4,86	0,012*
Cuadro clínico asociado						
con enfermedad intestinal						
Sí	272	23	8,4	1		
No	131	30	22,9	1,85	0,93-3,66	0,076
Prurito						
Sí	324	32	9,8	1		
No	79	21	26,6	2,11	1,02-4,37	0,044*
Lesiones en piel						
Sí	354	36	10,1	1		
No	49	17	34,7	2,98	1,32-6,70	0,008*

* Estadísticamente significativo

**Cuadro 8:** Resultados del diagnóstico molecular de *Dipylidium caninum* en pulgas *Ct. felis* y *Ct. Canis*, en perros domiciliados de la zona metropolitana de Toluca, México

	Muestras	Positivos	Prevalencia
*Ctenocephalides* felis	9	1	1,92 % (1/52)
ADN individual	24	3	5,76 % (3/52)
ADN en pools			
*Ctenocephalides canis*			
ADN individual	5	0	0 %
ADN en pools	14	1	1,92 % (1/52)
Total	52	5	9,61 % (5/52)

## Discusión

Se encontró una prevalencia de endoparásitos del 37,2 % en la población de perros domiciliados de la zona metropolitana de Toluca, cifra mayor a la reportada en otros estudios nacionales e internacionales de perros con atención médica: en Ciudad de México, las prevalencias registradas han sido de 20 % y 21,3 % ^9^^,^^29^, en Villahermosa, Tabasco, de 26,5 % ^8^, en sitios en Estados Unidos, de 12,5 % ^5^, en España, de 25 % ^30^, y en Austria, de 6 % ^6^.

La variación en la prevalencia y la intensidad se podría asociar con tres factores. El primero es el uso de cuatro técnicas de diagnóstico con diferentes propiedades de sensibilidad y especificidad, algunas de ellas más propicias para el diagnóstico de especies de parásitos ^31^^,^^32^. El segundo factor es la contaminación con parásitos en espacios públicos. En Toluca se analizaron siete parques y calles aledañas a estos, y se encontraron animales positivos para *Toxocara* spp. en todos los espacios muestreados ^33^. La presencia de *Toxocara* spp. en los espacios públicos en Toluca representa una fuente de reinfección para los animales y de posible transmisión al humano de la larva migrans visceral ^11^. El tercer factor es la falla en los protocolos de desparasitación; la población estudiada recibe atención médica y administración periódica de fármacos antiparasitarios, por lo que se esperaría una menor prevalencia. Esto podría indicar que, en los perros estudiados, no se están aplicando los antiparasitarios adecuados o su frecuencia de uso no es la correcta ^34^. En el presente estudio se sentarán las bases de un programa de control de estas parasitosis en los perros.

Se identificaron siete géneros o especies de parásitos, diversidad comparable con el rango reportado en estudios previos, el cual fue de 4 a 16 parásitos ^6^^,^^8^^,^^10^^,^^35^^,^^36^. En el 10,1 % de los perros se encontró parasitismo mixto, sobre todo de dos géneros o especies. En zonas tropicales de México, se han reportado mayores prevalencias de infecciones mixtas. En Yucatán, se reportó 21,3 % de parasitosis dobles y 3,1 % de triples ^2^; en Veracruz, se ha reportado 37,6 % de parasitosis dobles, 28,9 % de triples y 5,9 % de cuádruples ^37^; en Argentina y Rumania, la prevalencia de infección de parasitosis mixtas también ha sido mayor, con 16,8 % y 38 %, respectivamente ^28^^-^^36^. Al igual que en otros estudios, se observaron parasitosis mixtas de nematodos y protozoos ^26^^,^^36^^,^^38^. La importancia de conocer los géneros y las especies, así como las infecciones mixtas de parásitos, radica en lograr la selección correcta del fármaco antiparasitario, lo que permite tratamientos efectivos que ayuden a controlar estas parasitosis.

Los resultados muestran un predominio de géneros y especies de parásitos zoonóticos. Seis de las siete especies identificadas tienen potencial zoonótico, lo que coincide con otros estudios, como el de Ciudad de México, donde todas (100 %; 3/3) las especies reportadas fueron zoonóticas ^9^; el de Veracruz, con el 100 % (5/5) ^35^; el de Mérida, con el 75 % (3/4) ^10^, y el de Villahermosa, con el 85,7 % (6/7) ^8^. Este mismo comportamiento se registra a nivel mundial: en Austria, el 50 % (4/8) de los parásitos que afectan a los perros son zoonóticos ^6^; en Rumania, el 56,2 % (9/16) ^36^; en Polonia, el 71,4% (5/7) ^35^, y en Brasil, el 75 % (6/8) ^7^. La transmisión de estos parásitos al humano depende de distintos factores, pero el hecho de que se hayan identificado en animales que sirven de mascotas implica un mayor riesgo de transmisión debido al contacto estrecho con los humanos. Además, los perros positivos para estos parásitos que tienen acceso a espacios públicos como parques, calles o jardines, son una fuente de contaminación del ambiente ^11^^,^^39^.

El nematodo con mayor prevalencia fue *Toxocara* spp., con 16,6 %. En otras ciudades de México se reportan prevalencias variables, como en Mérida, 5,7 % y 6,2 % ^2^^,^^10^ y Ciudad de México, 6 % ^9^; en tanto que, en la ciudad de estudio, Toluca, se ha reportado en suelos y espacios públicos ^33^.

En el análisis multivariado de regresión logística, los perros menores de un año presentaron la mayor probabilidad de infección con *Toxocara* spp. Un hallazgo similar se ha reportado en España, Rumania y Argentina ^28^^,^^36^^,^^38^, donde los perros jóvenes son los más afectados por este nematodo, probablemente porque, durante la gestación y la lactancia, las larvas infectivas (L_3_) se reactivan y se transmiten a los cachorros por medio de la placenta y la leche ^40^. Asimismo, los animales jóvenes tienen una inmunidad poco desarrollada, lo que se refleja en una reacción inmunitaria insuficiente frente a los parásitos ^41^.

El segundo parásito con mayor prevalencia fue Giardia spp., con 13,4 %, la más alta reportada en perros domiciliados de México. En Villahermosa, Tabasco, se reportó en el 1 % de los perros ^8^, en tanto que, en estudios de perros callejeros, la prevalencia ha sido mayor: en Ciudad de México, 46,5 % ^42^ y en Veracruz, 42,6 % ^37^. En el 2008 en Australia, Palmer, et al., observaron un aumento en la prevalencia de este protozoario en perros y una tendencia a la disminución de helmintos, lo que asociaron al uso generalizado de antihelmínticos. Los perros con diarrea tenían 3,1 veces más probabilidades de tener parasitosis por Giardia spp.; este parásito daña las vellosidades del intestino delgado, lo que resulta en deficiencias en la absorción de nutrientes y aumento de la permeabilidad intestinal y, al final, genera destrucción de enterocitos ^43^. Lo mismo hallaron Bouzid, *et al*., en pacientes sintomáticos comparados con los asintomáticos ^44^.

El género *Ancylostoma* fue el tercero con mayor prevalencia en la población estudiada (9,2 %); además, es el parásito zoonótico con mayor prevalencia en los estudios de Ciudad de México (7,5 %) ^29^, Villahermosa, Tabasco (15,9 %) ^8^, Mérida, Yucatán (32,6 %) ^10^, Argentina (13,4 %) ^28^, Brasil (37,8 %) ^7^, Polonia (36 %) ^35^ y Rumania (33 %) ^36^.

Los perros menores de un año tienen 2,04 veces más probabilidades de infección con este nematodo, probablemente asociada con la transmisión lactogénica del parásito en los primeros días de vida ^40^. Asimismo, la permanencia en exteriores se asoció con la infección, probablemente porque los animales en contacto con el suelo en exteriores, patios y jardines son propensos a adquirir *Ancylostoma* spp. por vía oral y cutánea; llegan a ser parasitosis graves en el intestino delgado, donde producen los principales efectos en su fase adulta ^40^^,^^45^.

La diarrea y las manifestaciones clínicas intestinales también se asociaron con la infección de *Ancylostoma* spp. El nematodo se alimenta de la mucosa del intestino delgado y genera daño mecánico al adherirse a la misma mediante su cápsula bucal, y la diarrea suele acompañarse de sangre ^46^. Este parásito es uno de los más reportados en estudios internacionales y, según nuestros resultados, fue el que presentó más factores asociados con la infección.

El cestodo con mayor prevalencia fue D. caninum, con 4,7 %. La prevalencia de este parásito ha sido menor en estudios similares, como los llevados a cabo en Mérida, Yucatán (2,3 %) ^2^, en Villahermosa, Tabasco (0,3 %) ^8^, en Argentina (1,5 %) ^28^, en Rumania (1,4 %) ^34^, en Brasil (2,5 %) ^7^ y en Australia (0,1 %) ^47^.

La diarrea (OR=4,80) y las manifestaciones clínicas intestinales (OR=3,32) fueron factores asociados con la infección con *D. caninum*. El cestodo se adhiere a la pared intestinal por medio del escólex, lo que genera daño en la mucosa e inflamación del intestino ^46^; sin embargo, la diarrea se asocia poco con este parásito, cuyo síntoma característico es el prurito anal ^48^, el cual no se valoró en este estudio. La presencia de pulgas también fue un factor (OR=3,32) que favoreció la infección con *D. caninum*, ya que las pulgas *Ct. felis*, *Ct. Canis*, *Pulex irritans* y *T. canis* son huéspedes secundarios de este cestodo.

*Cystoisospora* spp. fue el segundo protozoo con mayor prevalencia (4,7 %), y la única especie encontrada que no se considera zoonótica. Este género se reporta en la mayoría de estudios epidemiológicos con prevalencias bajas comparadas con las de los nematodos u otros protozoos como *Giardia* spp. El 1,9 % de los perros estudiados en Mérida, Yucatán, resultaron positivos para *Cystoisospora* spp. ^10^; en Villahermosa, Tabasco, el 6,9 % ^8^; en Argentina, el 11,9 % ^28^; en Brasil el 3,5 % ^7^, y en Austria, el 2 % ^6^.

Los factores asociados con la infección por este género fueron la diarrea y las manifestaciones clínicas intestinales. El parásito destruye la lámina propia de todo el intestino delgado del perro hasta producir atrofia de las vellosidades. Se considera un patógeno primario de diarrea en animales jóvenes ^49^.

Otros parásitos identificados cuya prevalencia estuvo por debajo de 1 %, fueron *Taenia* spp. (0,74 %) y *T. vulpis* (0,24 %), ambas especies zoonóticas; el cestodo es el que posee mayor capacidad patógena en el humano ^50^, en tanto que *T. vulpis* solo se ha reportado como causante de zoonosis de manera excepcional ^51^. La poca prevalencia de este último contrasta con las altas cifras en otros estados de México o en estudios internacionales: Yucatán, 25,4 % y 5,7 % ^2^^,^^10^, Campeche, 9,2 % ^52^, Veracruz, 18,8 % ^37^, Brasil, 7,1 % ^7^, y Rumania, 16,6 % ^36^.

La prevalencia de ectoparásitos (13,1 %) fue menor a la reportada en otros estudios; en dos provincias de Brasil fue de 100 % y 89,7 % ^32^, y en Etiopía, 99,5% ^53^. Esta menor prevalencia se asocia con las características ambientales y de altitud en esta zona, las cuales no presentan cambios considerables entre las temporadas de lluvia y las secas. En la zona de estudio, el promedio de la temperatura es de 15 °C y la humedad del 70 % ^19^. A menor altitud, pero con temperatura y humedad mayores, los ciclos parasitarios se completan en un tiempo menor y puede haber hasta cuatro generaciones en un año ^53^, por lo cual, en zonas más elevadas, las prevalencias se incrementan.

Las pulgas fueron el ectoparásito más prevalente, 12,9 %, similar a lo reportado en Italia, 17,6 % ^12^. En el contexto nacional, en el estado de Aguascalientes, la prevalencia en perros domiciliados es de 12 %, cifra considerablemente menor de la encontrada en Yucatán, 48 %, o en Cuernavaca, 30,3 %. La especie predominante fue *Ct. felis*, lo que coincide con los reportes mencionados, con excepción del de Aguascalientes, donde *Ct. canis* fue la especie más prevalente ^13^^,^^15^^,^^54^^,^^55^.

Los perros con condiciones corporales deficientes presentaron mayor prevalencia de infección (OR=2,43). Debido a su hábito hematófago, los ectoparásitos pueden producir anemia y, en consecuencia, un déficit nutricional crónico ^46^. El prurito y las lesiones dérmicas se asociaron con las infestaciones por pulgas, con OR de 2,11 y 2,98, respectivamente. Al alimentarse, las pulgas inoculan saliva, la cual es muy alergénica, y ocasionan una dermatitis con intenso prurito; y al rascarse, los perros exacerban las lesiones en la piel ^55^.

Solo un perro presentó garrapatas de la especie *R. sanguineus*, la de mayor distribución en distintos estados de México ^16^^-^^18^. Este perro era originario de Yucatán, donde se reporta gran prevalencia de este ectoparásito ^18^, el cual es responsable de la transmisión de agentes como *Ehrlichia canis* y *Babesia canis*^56^.

*Trichodectes canis* fue el único piojo identificado en un perro. Este piojo no es común en perros; en México, Brazil y Etiopía, se reporta Heterodoxus spiniger como el piojo de mayor importancia en perros, con prevalencias del 2 al 67,4 % ^30^^,^^53^^,^^57^. Los resultados indican que los piojos, al igual que las garrapatas, no parasitan de manera importante a la población de perros de la zona de estudio.

En el presente estudio y mediante PCR, se verificó que el 9,6 % de las pulgas se encontraban infectadas con *D. caninum*, resultado similar al obtenido en países de Europa (8,3 %) y en Malasia (10 %). La especie de pulga con mayor prevalencia (5,7 %) de *D. caninum* fue *Ct. felis*, la más importante en la transmisión de este cestodo en Europa y Malasia ^26^^,^^58^. La importancia de esta especie radica en que es un parásito zoonótico cuya transmisión al humano ocurre principalmente en infantes que ingieren accidentalmente pulgas infectadas ^59^^-^^61^, por lo cual es importante un control de este vector en la región.

En conclusión, la prevalencia de endoparásitos en perros domiciliados de Toluca, México, se considera alta, dado que la población estudiada recibe atención médica periódica. Se observó un predominio de especies parasitarias con potencial zoonótico, lo cual puede representar un riesgo para los dueños de mascotas de la zona. Según el análisis de regresión logística, se debe hacer un diagnóstico parasitológico exhaustivo en los perros jóvenes, pues es el grupo con la mayor prevalencia de parásitos y que más cercanía tiene con los propietarios. También, se encontraron asociaciones de las parasitosis con la presencia de diarrea o semiótica intestinal, el tener acceso a espacios exteriores y la presencia de pulgas.

Por el contrario, la prevalencia de ectoparásitos en Toluca fue baja, siendo las pulgas las más prevalentes. La condición corporal, el prurito y las lesiones en piel, se asociaron con las infestaciones de ectoparásitos. La presencia de *D. caninum* en los perros y en las pulgas pone de manifiesto la importancia de un control integral de endoparásitos y ectoparásitos en la región, para disminuir las infecciones e infestaciones en los perros y reducir el riesgo de transmisión a los humanos

## References

[1] Dantas-Torres F, Otranto D (2014). Dogs, cats, parasites, and humans in Brazil: opening the black box. Parasit Vectors.

[2] Rodríguez-Vivas RI, Gutiérrez-Ruiz E, Bolio-González ME, Ruiz-Piña H, Ortega-Pacheco A, Reyes-Novelo E (2011). An epidemiological study of intestinal Darasites of dogs from Yucatán, México, and their risk to Dublic health. Vector Borne Zoonotic Dis.

[3] Baneth G, Thamsborg SM, Otranto D, Guillot J, Blaga R, Deplazes P (2016). Major parasitic zoonoses associated with dogs and cats in Europe. J Comp Pathol.

[4] Linardi PM, Santos JLC (2012). Ctenocephalides felis felis vs. Ctenocephalides canis (Siphonaptera: Pulicidae): Some issues in correctly identify these species. Rev Bras Parasitol Vet.

[5] Little SE, Johnson EM, Lewis D, Jaklitsch RP, Payton ME, Blagburn BL (2009). Prevalence of intestinal Darasites in Det dogs in the United States. Vet Parasitol.

[6] Hinney B, Gottwald M, Moser J, Reicher B, Schafer BJ, SchaDer R (2017). Examination of anonymous canine faecal samDles Drovides data on endoparasite prevalence rates in dogs for comparative studies. Vet Parasitol.

[7] Katagiri S, Oliveira-Sequeira TCG (2008). Prevalence of dog intestinal parasites and risk perception of zoonotic infection by dog owners in São Paulo State, Brazil. Zoonoses Public Health.

[8] Torres-Chablé OM, García-Herrera RA, Hernández-Hernández M, Peralta-Torres JA, Ojeda- Robertos NF, Blitvich BJ (2015). Prevalence of gastrointestinal parasites in domestic dogs in Tabasco, southeastern México. Rev Bras Parasitol Vet.

[9] Martínez-Barbabosa I, Gutiérrez M, Ruiz LA, Fernández AM, Gutiérrez EM, Aguilar JM (2015). Detección de Cryptosporidium spp. y otros parásitos zoonóticos entéricos en perros domiciliados de la Ciudad de México. Arch Med Vet.

[10] Ortega-Pacheco A, Torres-Acosta JFJ, Alzina-López A, Gutiérrez-Blanco E, Bolio-González ME, Aguilar-Caballero AJ (2015). Parasitic zoonoses in humans and their dogs from a rural community of tropical Mexico. J Trop Med.

[11] Otranto D, Dantas-Torres F, Mihalca AD, Traub RJ, Lappin M, Baneth G (2017). Zoonotic Parasites of sheltered and stray dogs in the era of the global economic and Political crisis. Trends Parasitol.

[12] Rinaldi L, Spera G, Musella V, Carbone S, Veneziano V, Iori A (2007). A survey of fleas on dogs in southern Italy. Vet Parasitol.

[13] Gracia MJ, Calvete C, Estrada R, Castillo JA, Peribáñez MA, Lucientes J (2008). Fleas parasitizing domestic dogs in Spain. Vet Parasitol.

[14] Costa-Junior LM, Rembeck K, Mendonça FL de M, Azevedo SC, Passos LMF, Ribeiro MFB (2012). Occurrence of ectoparasites on dogs in rural regions of the state of Minas Gerais, Brazil. Rev Bras Parasitol Vet.

[15] Bolio-González ME, Rodríguez-Vivas RI, Sauri-Arceo CH, Gutiérrez-Blanco E, Morales- Puerto F, Gutiérrez-Ruiz EJ (2012). Prevalencia de lesiones cutáneas de Ctenocephalides felis y Ctenocephalides canis en perros del estado de Yucatán, México. Bioagrociencias.

[16] Cruz-Vázquez C, García-Vázquez Z, Morales-Soto M (1998). Prevalence of Rhipicephalus sanguineus infestation in dogs in Cuernavaca, Morelos, México. Parasitol Día.

[17] Tinoco-Gracia L, Quiroz-Romero H, Quintero-Martínez MT, Rentería-Evangelista TB, González-Medina Y, Barreras-Serrano A (2009). Prevalence of Rhipicephalus sanguineus ticks on dogs in a region on the Mexico-USA border. Vet Rec.

[18] Rodríguez-Vivas RI, Apanaskevich DA, Ojeda-Chi MM, Trinidad-Martínez I, Reyes-Novelo E, Esteve-Gassent MD (2016). Ticks collected from humans, domestic animals, and wildlife in Yucatán, México. Vet Parasitol.

[19] INEGI, CONAPO Delimitación de las zonas metropolitanas de México.

[20] World Health Organization (WHO) (1991). Sample size determination in health studies: A practical manual.

[21] Bogel K, Frucht K, Drysdale G, Remfry J, Organization World Health, Unit Veterinary Public Health (1990). Guidelines for dog population management/preparation.

[22] Laflamme D (1997). Development and validation of a body condition score system for dogs. Canine Practice.

[23] Sirois M (2019). Laboratory manual for laboratory procedures for veterinary technicians.

[24] Rodríguez-Vivas RI, Cob-Galera LA (2005). Técnicas diagnósticas de parasitología veterinaria, Mérida, México.

[25] De Oliveira PR, Bechara GH, Denardi SE, Saito KC, Nunes ET, Szabó MPJ (2005). Comparison of the external morpholoay of Rhipicephalus sanguineus (Latreille, 1806) (Acari: Ixodidae) ticks from Brazil and Argentina. Vet Parasitol.

[26] Beugnet F, Labuschagne M, Fourie J, Jacques G, Farkas R, Cozma V (2014). Occurrence of Dipylidium caninum in fleas from client-owned cats and dogs in Europe usina a new PCR detection assay. Vet Parasitol.

[27] Boubaker G, Marinova I, Gori F, Hizem A, Müller N, Casulli A (2016). A dual PCR-based sequencina approach for the identification and discrimination of Echinococcus and Taenia taxa. Mol Cell Probes.

[28] Fontanarrosa MF, Vezzani D, Basabe J, Eiras DF (2006). An epidemioloaical study of gastrointestinal parasites of dogs from Southern Greater Buenos Aires (Argentina): age, gender, breed, mixed infections, and seasonal and spatial patterns. Vet Parasitol.

[29] Martínez-Barbabosa I, Gutiérrez-Cárdenas EM, Aguilar-Venegas J, Pimienta-Lastra R de J, Shea M (2011). Frecuencia de geohelmintos en canes domiciliados en siete delegaciones de la Ciudad de México. Vet Mex.

[30] Estrada-Peña A, Roura X, Sainz A, Miró G, Solano-Gallego L (2017). Species of ticks and carried pathogens in owned dogs in Spain: Results of a one-year national survey. Ticks Tick Borne Dis.

[31] Coelho WMD, Gomes JF, Amarante AFT do, Bresciani KDS, Lumina G, Koshino-Shimizu S (2013). A new laboratorial method for the diagnosis of gastrointestinal parasites in dogs. Rev Bras Parasitol Vet.

[32] Klimpel S, Heukelbach J, Pothmann D, Rückert S (2010). Gastrointestinal and ectoparasites from urban stray dogs in Fortaleza (Brazil): High infection risk for humans?. Parasitol Res.

[33] Romero-Núñez C, Yáñez-Arteaga S, Mendoza-Martínez GD, Bustamante-Montes LP, Ramírez-Durán N (2013). Contaminación y viabilidad de huevos de Toxocara spp. en suelo y heces colectadas en parques públicos, calles y perros en Toluca, México. Rev Cient (Maracaibo).

[34] ESCCAP (2017). Worm control in dogs and cats, Esccap.org.

[35] Bajer A, Bednarska M, Rodo A (2011). Risk factors and control of intestinal parasite infections in sled dogs in Poland. Vet Parasitol.

[36] Mircean V, Dumitrache MO, Mircean M, Colosi HA, Gvorke A (2017). Prevalence and risk factors associated with endoparasitic infection in dogs from Transylvania (Romania): A retrospective study. Vet Parasitol.

[37] Alvarado-Esquivel C, Romero-Salas D, Aguilar-Domínguez M, Cruz-Romero A, Ibarra-Priego N, Pérez-de León AA (2015). Epidemiological assessment of intestinal parasitic infections in doss at animal shelter in Veracruz, México. Asian Pac J Trop Biomed.

[38] Martínez-Carrasco C, Berriatua E, Gariio M, Martínez J, Alonso FD, De Ybáñez RR (2007). Epidemiological study of non-systemic parasitism in dogs in southeast Mediterranean Spain assessed by coprological and post-mortem examination. Zoonoses Public Health.

[39] Robertson ID, Irwin PJ, Lymbery AJ, Thompson RC (2000). The role of companion animals in the emergence of parasitic zoonoses. Int J Parasitol.

[40] Epe C (2009). Intestinal nematodes: Biology and control. Vet Clin North Am Small Anim Pract.

[41] Macpherson CNL (2013). The epidemioloqy and public health importance of toxocariasis: A zoonosis of global importance. Int J Parasitol.

[42] Ponce-Macotela M, Peralta-Abarca GE, Martínez-Gordillo MN (2005). Giardia intestinalis and other zoonotic parasites: Prevalence in adult dogs from the southern part of Mexico City. Vet Parasitol.

[43] Payne PA, Artzer M (2009). The biology and control of Giardia spp. and Tritrichomonas foetus. Vet Clin North Am Small Anim Pract.

[44] Bouzid M, Halai K, Jeffrevs D, Hunter PR (2015). The prevalence of Giardia infection in dogs and cats, a systematic review and meta-analysis of prevalence studies from stool samples. Vet Parasitol.

[45] Traversa D, Franqipane di Regalbono A, Di Cesare A, La Torre F, Drake J, Pietrobelli M (2014). Environmental contamination by canine geohelminths. Parasit Vectors.

[46] Georgi JR, Georgi ME (1994). Parasitología en clínica canina.

[47] Palmer CS, Thompson RCA, Traub RJ, Rees R, Robertson ID (2008). National study of the gastrointestinal parasites of dogs and cats in Australia. Vet Parasitol.

[48] Conboy G (2009). Cestodes of dogs and cats in North America. Vet Clin North Am Small Anim Pract.

[49] Mitchell SM, Zajac AM, Charles S, Duncan RB, Lindsav DS (2007). Cystoisospora canis Nemeséri, 1959 (syn. Isospora canis), infections in dogs: Clinical signs, pathogenesis, and reproducible clinical disease in beagle dogs fed oocysts. J Parasitol.

[50] Robertson ID, Thompson RC (2002). Enteric parasitic zoonoses of domesticated dogs and cats. Microbes Infect.

[51] Márquez-Navarro A, García-Bracamontes G, Álvarez-Fernández BE, Ávila-Caballero LP, Santos-Aranda I, Díaz-Chiguer DL (2012). Trichuris vulpis (Froelich, 1789) infection in a child: A case report. Korean J Parasitol.

[52] Encalada-Mena LA, Duarte-Ubaldo EL, Vargaz-Magaña JJ, García-Ramírez MJ, Medina-Hernández RE (2011). Prevalence of gastroenteric parasites of dogs in the city of Escárcega.

[53] Kumsa BE, Mekonnen S (2011). Ixodid ticks, fleas and lice infesting dogs and cats in Hawassa, southern Ethiopia. Onderstepoort J Vet Res.

[54] Cruz-Vázquez C, Castro-Gámez E, Parada-Fernández M, Ramos-Parra M (2001). Seasonal occurrence of Ctenocephalides felis felis and Ctenocephalides canis (Siphonaptera: Pulicidae) infesting dogs and cats in an urban area in Cuernavaca, México. J Med Entomol.

[55] Hernández-Valdivia E, Cruz-Vázquez C, Ortiz-Martínez R, Valdivia-Flores A, Quintero- Martínez MT (2011). Presence of Ctenocephalides canis (Curtis) and Ctenocephalides felis (Bouché) Infesting dogs In the city of Aguascalientes, México. J Parasitol.

[56] Dantas-Torres F (2010). Biology and ecology of the brown dog tick, Rhipicephalus sanguineus. Parasit Vectors.

[57] Torres-Chable OM, Baak-Baak CM, Ciqarroa-Toledo N, Zaraqoza-Vera CV, Ariona-Jiménez G, Moreno-Pérez LG (2017). First report of chewing lice Heterodoxus spiniger (Enderlein, 1909) and Trichodectes canis (De Geer, 1778) on domestic dogs at Tabasco, México. Southwest Entomol.

[58] Low VL, Prakash BK, Tan TK, Sofian-Azirun M, Anwar FHK, Vinnie-Siow WY (2017). Pathogens in ectoparasites from free-ranging animals: Infection with Rickettsia asembonensis in ticks, and a potentially new species of Dipylidium in fleas and lice. Vet Parasitol.

[59] Traversa D (2013). Fleas infesting pets in the era of emerqinq extra-intestinal nematodes. Parasit Vectors.

[60] Neira O P, Jofré M L, Muñoz S N (2008). Infección por Dipylidium caninum en un preescolar: presentación del caso y revisión de la literatura. Rev Chilena Infectol.

[61] Narasimham MV, Panda P, Mohanty I, Sahu S, Padhi S, Dash M (2013). Dipylidium caninum infection in a child: A rare case report. Indian J Med Microbiol.

